# Supplementary material for: Comparison of Marker Effects and Breeding Values at Two Levels at THI for Milk Yield and Quality Traits in Brazilian Holstein Cows
Source: Genes (Basel). 2022 Dec 21;14(1):17. doi: 10.3390/genes14010017 (PMC9858941; doi:10.3390/genes14010017)
Supplement: Supplementary file 1 [file genes-14-00017-s001.zip › genes-2024204-supplementary.pdf]

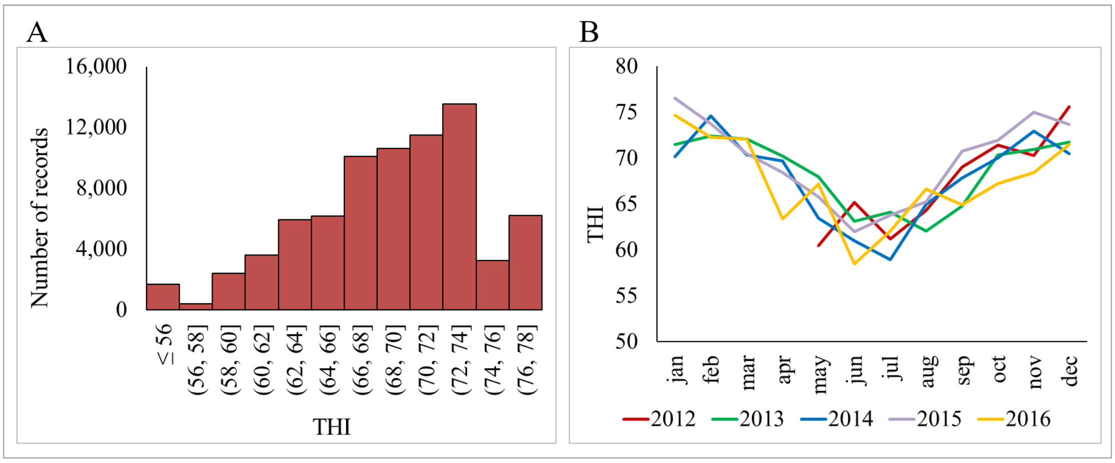

Supplementary Figure S1. (A) Number of records per THI and (B) distribution of mean THI per month (taken from Carrara et al., 2021, doi:10.1111/jbg.12636).
